# The Impact of Palliative Care and Nursing Intervention on the Psychology and Quality of Life of Elderly Patients with Colorectal Cancer

**DOI:** 10.1155/2022/7777446

**Published:** 2022-06-13

**Authors:** Ai Zhang, He Fu

**Affiliations:** ^1^Department of Geriatric Intensive Care Unit Nursing, Sichuan Provincial People's Hospital, University of Electronic Science and Technology of China, Chengdu 610072, China; ^2^Sichuan Translational Medicine Research Hospital, Chinese Academy of Sciences, Chengdu 610072, China

## Abstract

Colorectal cancer patients face physical, psychological, and social difficulties. Psychosocial therapies appear to be successful in improving cancer patients' psychological and social results. Preoperative and postoperative psychological therapies for colorectal surgery patients have not been extensively studied. During their treatment, up to 35% of cancer patients experience clinically severe psychological discomfort. As a consequence, a greater knowledge of health-related quality of life and its causes can assist oncology nurses in developing effective treatments to improve the health-related quality of life. The palliative care model and the nursing intervention model are used in this study to assess the effectiveness of an individually customized nursing intervention for lowering chemotherapy-related symptom distress in adult patients with colorectal cancer. Initially, the dataset is collected and split into the control group and the experimental group. The patient conditions are evaluated using the novel accelerated gradient boosting regression tree (AGBRT) estimation model. For improving the evaluation process, we have proposed the enriched gravitational search optimization algorithm (EGSOA). The system's success is evaluated in terms of the patients' psychological well-being and quality of life.

## 1. Introduction

Colorectal cancer is one of the leading causes of cancer-related death worldwide, accounting for an estimated 1,033,197 new cases and 520,902 deaths every year [1]. A person's quality of life is determined by a variety of elements, including (a) physical considerations, (b) psychological aspects, (c) social factors, (d) spiritual factors, and (e) cultural factors. Because of the intricacy of the factors that have an influence on one's quality of life, there are objective and subjective elements to it. Reference [2] refers to subjective well-being in the context of these factors, taking into account how each patient physically and psychologically felt at the time of the examination. In addition to a reluctance to return to work after surgery, avoiding social connections, and modifying their leisure activities, individuals with colorectal cancer may choose hobbies that allow for passive role-playing or limited contact with other people, according to the American Cancer Society. Patients who have colorectal cancer may find it more difficult to cope with their symptoms than people not having it. One of the most critical aspects of a patient's adjustment to a colostomy is their need for stoma care instruction. Individualized teaching and psychological support in the acceptance of their new body image may help patients better adjust to having a permanent colostomy. When cancer patients have stomas, their sexuality is also affected. Many stoma sufferers have lifestyle changes and sexual difficulties, according to [3]. Colorectal cancer patients who have had surgery are more likely to suffer from erectile dysfunction if they have a stoma. Additionally, patients with stomas must deal with the aftermath of a major surgery, the loss of a critical biological function, a change in their self-perception, and modifications to their physical well-being and self-care requirements. A study comparing stoma patients to those without one found that the nonstoma group was happier, had fewer changes in body image, and was more likely to return to work. Colorectal cancer patients who had surgery were shown to suffer a significant decrease in quality of life, regardless of whether or not they needed a colostomy. The low self-esteem and physical changes that stoma patients have to deal with lead to greater rates of depression, loneliness, and despair among them, particularly among young women. Patients with cancer, particularly those who share a home with them, experience a wide range of emotional and cognitive effects as well as physical ones as a result of their condition. Caregiving for a family member may put a strain on a person's ability to bounce back, causing them to believe that their physical, social, emotional, and spiritual well-being is deteriorating as a result of the stress. This disease has a profoundly unpleasant impact on the patient and family, often resulting in feelings of worry and depression as well as a sense of helplessness and despair.

Researchers discovered that spouses suffer significantly more emotional pain than patients during the first year after a cancer diagnosis as a result of witnessing their partner's suffering and not knowing how to help. This discovery helped them gain a better understanding of the impact of cancer on the family. When it comes to gender, when the caregiver is a woman, especially if she is the wife, the discomfort level might be greater. Illness has an impact on marital relationships as well. These impacts are stronger in stoma patients, owing to the stoma-induced alterations in body image. It is the performance of health experts that determines whether or not patients would accept colostomy specialized care training and whether or not they will provide unconditional support to the patients' family who is also dealing with sickness. Sexuality is also affected by the condition, with considerable alterations, especially in male patients who have had colostomy surgery, with resulting nerve damage to the genital organs, resulting in erection and ejaculation failure. An abdominoperineal excision preserves most of these nerve systems in female patients, enabling sexual function to be preserved. Nonetheless, psychological variables have a significant influence in changes in sexual life. Patients want to be treated with decency and respect as individuals. They want access to the most effective therapies and the ability to make informed decisions about their treatment and care. As a result, if every health care worker provides genuine support to the patient and family, it is feasible to achieve a considerable increase in patient satisfaction, as well as a favourable influence on the patient's adaptation to disease and, as a result, on his or her quality of life. The study participants with colorectal cancer who were undergoing chemotherapy and those with chronic liver disease, diabetes, and other diseases were excluded. The following sections are arranged as follows: related work is presented in Section 2; methods and materials are presented in Section 3; results and discussion are presented in Section 4; the conclusion of Section 5 is presented here.

## 2. Related Work

In [4], the study participants with colorectal cancer who were undergoing chemotherapy in metropolitan Brazil were surveyed about their perceptions of stress, social support, and resilience [5]. While cancer patients in Jordan were undergoing treatment, the researchers wanted to look at the relationship between hope, melancholy, anxiety, and health-related quality of life in order to better understand their experiences [6]. Cancer patients are confronted with a variety of emotional, mental, social, and spiritual difficulties. Nothing, however, has been done to address these criteria as of yet [7]. To investigate if there are disparities between men and women when it comes to assessing the quality of life in colorectal cancer patients after treatment, 144 patients (72 men and 72 females) were included in this cross-sectional research, which took place at a hospital in southern Brazil and involved chemotherapy. It is the purpose of this review to summarize the clinical and pathological characteristics associated with CRC in older patients, as well as optical surgical procedures such as laparotomy excision, endoscopic, and chemotherapy strategy [8, 9]. The researchers wanted to see if a 4-week preoperative trimodal prehabilitation program that included exercise, nutritional supplements, and counseling services on relaxation exercises was strong enough to change workout activities to improve the functional ability in geriatric people who were scheduled to undergo colon cancer surgery [10]. Colorectal cancer (CRC) is more prevalent in older people, and surgery is the most common kind of therapy. It was our goal in this randomized controlled study to explore if preoperative GA-guided treatment might minimize the incidence of postoperative problems in individuals who had a history of fragile colorectal cancer [11]. The study's purpose was to examine whether colorectal cancer patients' quality of life differed from the quality of life of the general population in relation to changes in work involvement during a 12-month period. The American Cancer Society collected data on non-Hispanic black (blacks) and non-Hispanic white (whites) patients aged 18–64 who were diagnosed with a single or first primary invasive stage I–IVCRC from 2004 to 2012 [1, 12]. A search in health-related databases yielded no results. First, we filtered out articles based on their titles and abstracts using the exclusion criteria. At the end of the process, eight studies remained. Next, we analysed the data from the selected studies and assessed their methodological integrity [13]. Periodontal diseases, such as tooth loss, may exacerbate inflammation in the body, and immunological dysregulation and changes in the microbiota of the gut may have an impact on colorectal carcinogenesis. Only a few epidemiological studies have looked at the connection between gum disease and the risk of colorectal cancer. When it came to periodontal diseases and the number of natural teeth, the Nurses' Health Study had it covered. Hospital nurses' perspectives and experiences in evaluating the needs of colorectal cancer survivors and patients in rehabilitation after their diagnosis and treatment are examined in this study [14, 15]. “CRISP,” a colorectal cancer risk prediction tool, was created and optimized for primary care usage in this study. It is the purpose of CRISPR tool to encourage risk-adjusted screenings for colon cancer [16]. Colorectal cancer chemotherapy patients need an educational nursing intervention that enables them to take responsibility for their own treatment and well-being. In this therapy, quality of life and nutritional hazards are not primary outcomes. Inpatients with CRC and lifestyle modifications may help increase cancer survivability, but therapy options are getting increasingly complex and long, which may limit the availability of these treatments [17, 18]. Colorectal cancer screening has been shown to minimize the mortality rate from the illness. Screening disparities remain among ethnic minorities, particularly African Americans [19]. A classifier for distinguishing between benign and malignant tumours is proposed in this study. In this study, nurses treated 50 patients with advanced-stage lung cancer in two separate groups, one as a control group and the other as a treatment group. Control and test groups are established in [20]. Patients in the control group get standard nursing care whereas those undergoing thoracic surgery are exposed to a fast rehabilitative nursing intervention. The patients' progress during rehabilitation is tracked using real-time data gathering as well as statistical analysis and follow-up surveys in this study [21]. There were two groups of patients in the study: those who got a supportive care assessment in person and those who received regular care practices or a standard nurse intervention. It was required that participants be diagnosed within seven days after their surgery and had previously not received therapy for cancer [22, 23]. The use of patient-friendly colorectal capsule endoscopy in large populations might help colon cancer screening (CCE). In order for it to be widely deployed, more research must be done first. In [24], the microbiota of the gastrointestinal tract (GI tract) may be implicated in the pathogenic process. Through an imbalanced microbiota and an impaired immune system, GI microbiota dysbiosis has been linked to respiratory illnesses, including COVID-19, as well as sporadic colorectal cancer (CRC) response. It is critical to comprehend the potential role of probiotics in microbial stability sustaining the respiratory and gastrointestinal tract integrity in SARS-CoV-2 infected people with dysbiosis and the development of colorectal cancer [25]. Recommending hyaluronic acid rectal enemas, administered daily, might have a role in the prevention of proctitis in patients undergoing curative static IMRT to the prostate or the prostate bed, not having proved inferior to the daily beclomethasone dipropionate suppositories regarding rectal toxicity rates but being able to reduce its severity [26]. Detailed Allium vegetables and their constituents have recently been extensively investigated due to the numerous possible beneficial properties, establishing them as an additional treatment modality in different gastrointestinal cancers including gastric, colon, liver, esophageal, and pancreatic cancer. According to the epidemiological evidence, as well as many in vivo and in vitro studies, the abovementioned substances seem to be effective in the prevention and inhibition of the progression of carcinogenesis. Due to high concentrations of organosulfur compounds, which exhibit anticarcinogenic, antimicrobial, as well as anti-inflammatory properties, Allium constituents are believed to constitute a promising prevention and supportive therapy for oncological patients. Besides, it was demonstrated that a combination of Allium extracts with chemotherapy provided satisfactory clinical outcomes while at the same time being cost-effective.

## 3. Materials and Methods

**Figure 1:**
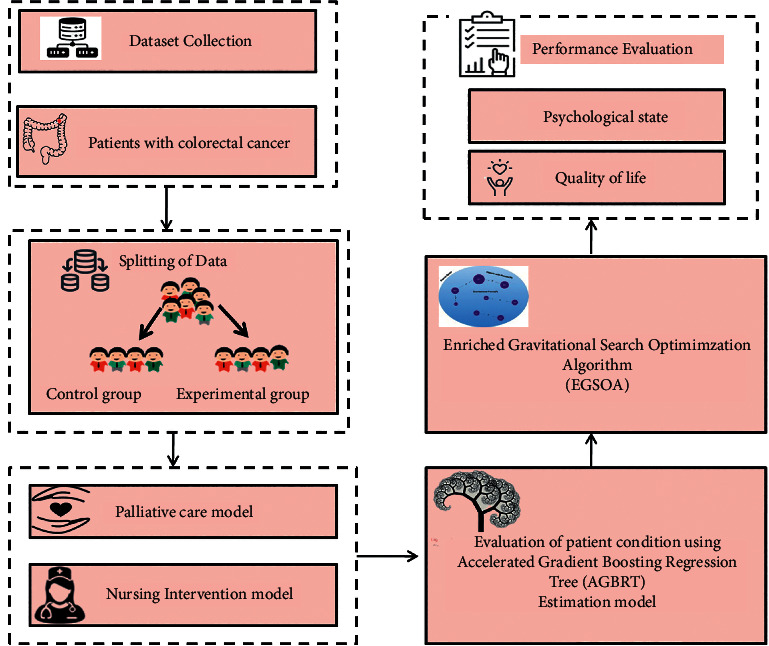
Proposed system's schematic flow.

The main aim of this article was to investigate the effectiveness of an individually customized nursing intervention for lowering chemotherapy-related symptom distress in adult patients with colorectal cancer; this article employs the palliative care model and the nursing intervention model. Initially, the dataset is gathered and separated into a control group and an experimental group. The patient conditions are assessed utilizing the innovative accelerated gradient boosting regression tree (AGBRT) estimate technique. For enhancing the assessment process, we have suggested the enriched gravitational search optimization algorithm (EGSOA). The overall flow diagram is shown in Figure 1.

### 3.1. Dataset Collection

A total of 60 colorectal cancer patients receiving colostomy in the major department of surgery of Changsha's Fourth Hospital were recruited as study participants between 2018 August and 2020 February, including 32 men and 28 women [23]. They were split into two groups: (i) an experimental group with 30 patients and (ii) a control group with 30 patients. The research comprised colorectal cancer patients. Those with severe cardiopulmonary or other serious organ problems, as well as patients with consciousness difficulties, communication abnormalities, or surgical contraindications, were excluded from the study. All of the patients volunteered to take part in the study and completed an informed consent form. Patients with colostomies had their health outcomes and psychological distress evaluated(i) before, (ii) during, and (iii) after the procedure; the patient's overall quality of life is evaluated with (a) age, (b) gender (c) pathogenic region, and (d) pathological patterns; the degree of education did not differ significantly between the two groups (*P* > 0.05), indicating similarities. More details are found in Table 1.

### 3.2. Splitting of Data

The dataset is separated into two groups: the experimental group and the control group. Each of the two groups consisted of 30 patients: 30 in the experimental group and 30 in the control group. There were no intervention programs for the control group patients. The patients in the study were treated according to the palliative care paradigm.

### 3.3. Nursing Intervention

#### 3.3.1. Control Group

The patients in the control group received standard medical treatment. By having a supervising physician present throughout the procedure, the patient's comfort and unique surgical procedures are taken into consideration. The patients' illnesses and colostomies, dietary adjustments, and emotional support are among the other unique therapies. As a result, patients are able to actively participate in their treatment by receiving thorough answers to relevant queries and postoperative care instructions. There was a palliative healthcare strategy for patients in the experimental group.

#### 3.3.2. Experimental Group

Measures taken to ensure that patients were receiving a palliative care model included the following:

- Patients were asked about their preferences for stoma placement, and nurses worked with doctors to find a location that would not interfere with the patients' ability to dress comfortably. The nursing staff worked with doctors to locate the stoma's location before surgery, and they discussed this with the enterstomal therapist, surgeons, and patients one day before surgery.
- Medical professionals kept a tight eye on their patients' vital signs following surgery, notifying the doctor right away if anything seemed out of the ordinary with their vital signs
- After a colostomy, patients typically experience anxiety, despair, and other undesirable feelings. In order to better serve patients, the nursing staff had to learn to speak gently, encourage patients to express their emotions, and listen patiently to them. A colostomy awareness education program was also required for patients. Colostomy nursing procedures and functions were taught to patients. The patients' fear and anxiety were alleviated and their self-esteem was built up as a result of the nursing staff's employment of culturally and knowledge-appropriate methods of publicizing this information.
- Ostomy bag usage, replacement, and monitoring procedures were taught to the patients and their families by the nursing staff, and the patients and families were instructed to monitor for signs of edema, ischemia necrosis, and the related therapeutic measures at colostomy.
- Patients' self-care abilities are improved; the nurses' explanations of how to utilise the ostomy bag may help patients to not only avoid infection and strange odours, but also boost their self-esteem and lessen their fear about engaging in social activities following surgery.
- The word “quality of life” refers to a broad and comprehensive concept that encompasses subjective appraisals of life's benefits and drawbacks. It encompasses characteristics such as overall functioning and well-being, as well as a person's appraisal of their own experiences and mental and interpersonal health. Quality of life principles vary by field and are usually complex and difficult to put into practice. Due to a significant impact on the aesthetic look of the abdomen and physical performance, patients with a distinctive stoma encounter many challenges in terms of their quality of life.

### 3.4. Evaluation of Patient Condition Using Accelerated Gradient Boosting Regression Tree (AGBRT)

**Figure 2:**
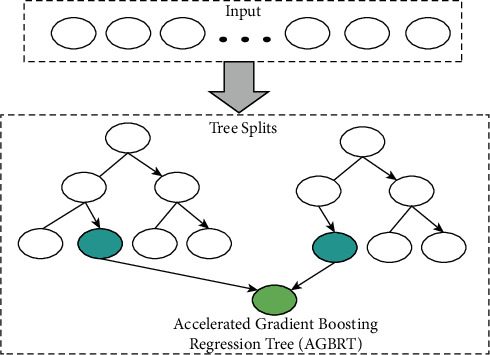
Colorectal cancer analysis using the accelerated gradient boosting regression tree technique.

This article proposes the accelerated gradient boosting regression tree technique, which uses regression trees with the same decision criteria as decision trees. Regression and classification are both supported. This approach is a scalable and efficient form of the accelerated gradient boosting machine (AGBM), which has been used in computer vision, data mining, and other domains. The accelerated gradient boosting regression tree technique is primarily enhanced in two areas: the tree building speed and the proposal of a novel distributed technique for tree searching, as illustrated in Figure 2.

The goal function is provided by the equation, assuming the regression tree model consists of *R* decision trees

(1) $$\begin{array}{c} {\hat{x}}_{i}=\sum_{r=2}^{r}g_{r}\left(z_{i}\right),\;g_{r}\in F\left(1\right), \end{array}$$

where *G* represents the space of the regression tree model, and each corresponds to an independent tree with leaf scores. The following equation expresses the loss function:

(2) $$\begin{array}{c} L_{s}\left(g_{p}\right)=\sum l_{s}\left({\hat{x}}_{i},x_{i}\right)+\sum \beta \left(g_{p}\right), \end{array}$$

where *l*_*s*_ denotes differentiable loss function, ${\hat{x}}_{i}$ denotes predicted output, *x*_*i*_ denotes true output, and *l*_*s*_ measures variation between ${\hat{x}}_{i}$ and *x*_*i*_.

Regularization terms penalise the model's complexity in an effort to prevent it from being too well fitted.

Here, *β* and${\hat{x}}_{i}$ can be expressed as follows:

(3) $$\begin{array}{c} {\hat{x}}_{i}^{\left(p\right)}={\hat{x}}_{i}^{\left(p-1\right)}+g_{p}\left(z_{i}\right),\\ \beta \left(f\right)=\gamma P+\frac{1}{2}{\left|\left|W\right|\right|}^{2}, \end{array}$$

where *P* denotes leaf nodes and *W* represents values of every leaf.

As a result, we may deduce that

(4) $$\begin{array}{c} L_{s}\left(g_{p}\right)\approx \sum_{j=1}^{P}\left[\left(\sum_{i\varepsilon I_{j}}s_{i}\right)w_{j}+\frac{1}{2}\left(\sum_{i\varepsilon I_{j}}h_{i}+\lambda \right)W_{j}^{2}\right]+\gamma P, \end{array}$$

where *s*_*i*_ and *h*_*i*_ are the loss function's first-order and second-order gradient statistics, respectively. To regulate the degree of regularisation, the parameters are constants. They are employed to keep overfitting at bay.

### 3.5. Enriched Gravitational Search Optimization Algorithm (EGSOA)

The enriched gravitational search optimization algorithm is a novel optimization approach that depends on component interaction to achieve comprehensive information transmission. Newton's law states that there is universal gravitation. Each component (mass) in EGSOA may be considered as a moving item with a mass that is interacting with the rest of the system. A component with a great mass has an enhanced area, which results in a higher fitness value. Assume there are *N* components, ds represents dimension gap, and component k's location is as follows:

(5) $$\begin{array}{c} o_{k}=\left(o_{k}^{1},o_{k}^{2},\ldots ,o_{k}^{ds}\right). \end{array}$$

The location of component *k* in the *ds* dimension is represented by ods, and the solution may be deduced from the location of each component. The force exerted by component *j* on component *k* is as follows:

(6) $$\begin{array}{c} b_{kj}^{ds}=H\left(q\right)\times \frac{M_{k}\left(q\right)\times M_{k}\left(q\right)}{R_{kj}+cs}\times \left(o_{j}^{ds}\left(q\right)-o_{k}^{ds}\left(q\right)\right). \end{array}$$

The inertial masses of components *k* and *j* are *M*_*k*_ and *M*_*j*_, respectively. H (*t*) represents gravitational steady, *cs* is a tiny steady to avoid division by zero mistakes, and *R*_*kj*_ represents the Euclidean distance between component *k* and component *j*:

(7) $$\begin{array}{c} H\left(t\right)=H\left(0\right)\times {\left(\frac{q}{q_{\max}}\right)}^{\alpha }, \end{array}$$

where *H*(0) denoted is gravity's starting value. *q*_max_ denotes highest number of iterations, *α* < 1.

In the *ds* dimension, component k's resulting force and acceleration are described as follows:

(8) $$\begin{array}{c} B_{k}^{ds}\left(q\right)=\sum_{j=1,j\neq k}^{N}r_{j}\times B_{kj}^{ds}\left(q\right), \end{array}$$

where *r*_*j*_ represents a random value between (0, 1) and *B*_*k*_^*ds*^(*q*) represents the gravity value of component *j* to component *k*.

(9) $$\begin{array}{c} c_{k}^{ds}\left(q\right)=\frac{B_{k}^{ds}\left(q\right)}{M_{k}\left(q\right)}. \end{array}$$

The following is the updated formula for component *k*'s velocity and location in the *ds* dimension:

(10) $$\begin{array}{c} y_{k}^{ds}\left(q+1\right)=r_{j}\times y_{k}^{ds}\left(q\right)+c_{k}^{ds}\left(q\right),\\ P_{k}^{ds}\left(q+1\right)=P_{k}^{ds}\left(q\right)+y_{k}^{ds}\left(q+1\right), \end{array}$$

where *y*_*k*_^*ds*^(*q*) and *P*_*k*_^*ds*^(*q*)denote the velocity and position of the *k* component, respectively. The randomization may be increased using *r*_*j*_.

The fitness value determines the inertial mass of components. The bigger the amount of inertia, the closer you are to the ideal solution.

(11) $$\begin{array}{c} m_{k}\left(q\right)=\frac{{\text{fitness}}_{k}\left(q\right)-\text{fitworst}\left(q\right)}{\text{fitbest}\left(q\right)-\text{fitworst}\left(q\right)},\\ M_{k}\left(q\right)=\frac{m_{k}\left(q\right)}{\sum_{j=1}^{N}m_{j}\left(q\right)}. \end{array}$$

We have randomly initialized the population's location in discrete space with the numbers 0 and 1. The transfer probability functions are

(12) $$\begin{array}{c} T\left(y_{k}^{d}\left(q\right)\right)=\left|\text{tang}\left(y_{k}^{d}\left(q\right)\right)\right|,\\ o_{k}^{d}\left(q+1\right)=1-o_{k}^{d}\left(q\right),\\ o_{k}^{d}\left(q+1\right)=1-o_{k}^{d}\left(q\right). \end{array}$$

When the random value *r* is smaller than *T*(*y*_*k*_^*d*^(*q*)), the component will travel in the following manner.

## 4. Result and Discussion

This section goes into the behavioral analysis of the recommended technique. This section also discusses the effects of the recommended palliative care models on the psychological states, and quality of life. The experimental and control groups' findings are compared and evaluated. We selected four time periods for the whole investigation. The period preceding the intervention is denoted by the letter *T*1. The discharge time is indicated by the letter *T*2. *T*3 stands for three months after discharge. *T*4 denotes a period of six months after discharge.

### 4.1. Quality of Life

**Figure 3:**
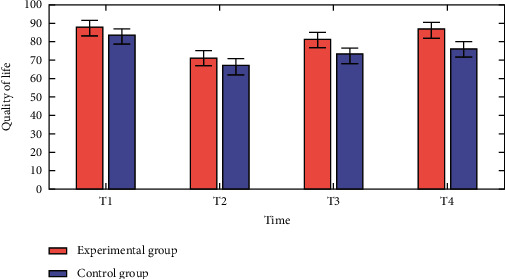
Comparative analysis of quality of life vs time.

In order to assess the patients' quality of life, the Quality of Life Scale was used. Life quality of primary care did not change significantly across the groups before and after intervention (*p* > 0.050). At three and six months after discharge, the life quality of those in the intervention group was significantly higher than that of the control group. This improvement persisted beyond the intervention. According to the results shown in Figure 3, the intervention group's quality of life improved significantly when the intervention time point was extended, reaching its peak 3–6 months after discharge, with a significant difference in the trend of time variation between the control and experimental groups. Physical performance, physical role performance, general health impressions, energy, emotional role performance, and mental health scores were all significantly poorer in the experimental group compared to the control group as shown in Table 2.

### 4.2. Psychological State

**Figure 4:**
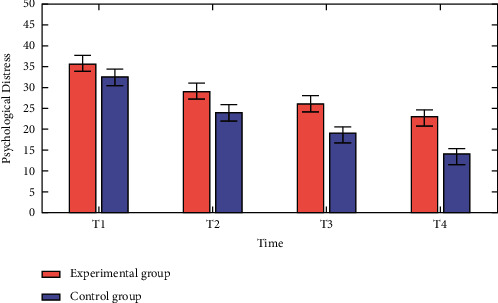
Comparative analysis of psychological distress vs time.

The psychological distress of the patients differed between the two groups (*p* 0.05), there was no difference before and after the intervention, and communication between time and group demonstrated that various groups had distinct temporal constraints. The movement of various intervention time points following the intervention reduced psychological discomfort, and the intervention group significantly reduced 6 months after discharge; the trend of the intervention time shift revealed a significant difference between the control and experimental groups, as shown in Figure 4 and the psychological states of the control and experimental groups were compared as shown in Table 3.

### 4.3. Comparison of Negative Emotion Scores

**Figure 5:**
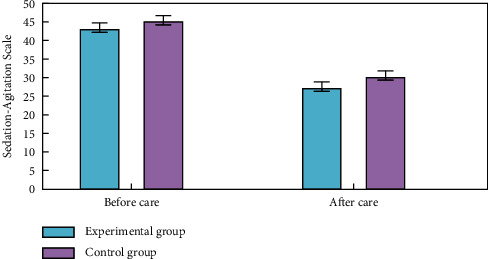
Comparative analysis of negative emotion scores of SAS (Sedation-Agitation Scale).

**Figure 6:**
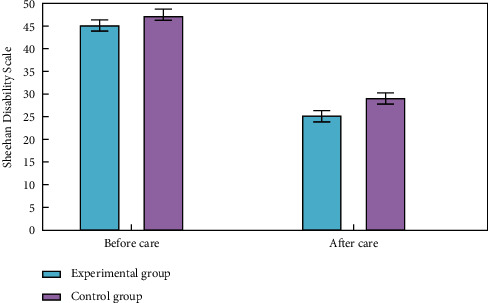
Comparative analysis of negative emotion scores of SDS (Sheehan Disability Scale).

To examine the patients' psychological negative feelings, we employed the Riker for both (a) SAS (Sedation-Agitation Scale) and (b) SDS (Sheehan Disability Scale). There was no significant difference between the two groups' SAS and SDS readings before treatment. Aftercare decreased negative emotion ratings considerably in both groups compared to before, with the experimental group demonstrating a higher improvement. Statistically, the differences were substantial. More details can be seen in Figures 5 and 6.

## 5. Conclusion

The impact of the palliative care model nursing intervention programs on the psychological state and life quality of elderly colorectal patients is investigated in this study using the accelerated gradient boosting regression tree approach. The data were evaluated using Spearman's correlation analysis and hierarchical correlation analysis. Palliative care models can not only improve caregivers' capacity to care for sick people with permanent colostomies, reduce mental harm, and improve liveability; they can also reduce the risk of complications associated with permanent colostomies and increase their flexibility, providing a conceptual underpinning and reference for the caregivers' care methods during the recovery phase of illness. However, since the suggested model is still in its early phases of development, subsequent research will need to increase the sample size and prolong the inquiry in order to further evaluate the treatment program's efficacy.

## Figures and Tables

**Table 1:** Data.

Factors		Experimental group (*n* = 30)	Control group (*n* = 30)	*X* ^2^	*p*
Age	≤61	14 (44.67)	15 (50.10)	0.289	0.655
	≥61	16 (57.78)	15 (49.50)		
Gender	Male	16 (53.80)	17 (57.68)	0.087	0.799
	Female	14 (46.79)	13 (44.59)		
Pathogenic region	Colon cancer	15 (50.50)	13 (44.67)	0.285	0.655
	Rectal cancer	15 (49.50)	17 (57.59)		
Pathological pattern	Squamous carcinoma	17 (56.87)	16 (53.68)	0.068	0.815
	Glandular cancer	13 (43.78)	14 (46.90)		
BMI (kg/m^2^)	≤23	18 (60.80)	16 (53.60)	0.288	0.648
	≥23	12 (40.68)	14 (47.78)		
Educational level	Below high school	20 (68.78)	21 (70.39)	0.785	0.7891
	High school and above	10 (33.69)	9 (30.48)		
Smoking history	Present	14 (47.58)	13 (43.75)	0.068	0.815
	Absent	16 (54.76)	17 (56.48)		

**Table 2:** Comparative analysis of quality of life.

Quality of life	Group	*n*	Learn	*p*
Physical functioning	Control	30	64.56	0.020
	Experimental	30	36.49	

Physical role	Control	30	71.58	0.020
	Experimental	30	30.25	

Body pain	Control	30	47.32	0.140
	Experimental	30	54.98	

General health	Control	30	63.91	0.020
	Experimental	30	39.41	

Vitality	Control	30	61.21	0.020
	Experimental	30	41.81	

Social functioning	Control	30	56.15	0.490
	Experimental	30	45.76	

Emotional role	Control	30	68.52	0.020
	Experimental	30	33.98	

Mental health	Control	30	59.11	0.040
	Experimental	30	43.12	

**Table 3:** Comparative analysis of psychological conditions.

Psychological states	Experimental group (*n* = 30)	Control group (*n* = 30)	*p*
The total score of the beck depression inventory (mean ± SD)	15.7 ± 9.0	13.6 ± 10.1	0.90
The total score of the beck anxiety inventory (mean ± SD)	24.2 ± 5.2	15.1 ± 7.5	0.02
Health concerning sexual performance	9.0 (16%)	13.0 (24%)	
Arizona Sexual Experiences Scale (mean ± SD)	55.9 ± 12.4	46.3 ± 11.90	

## Data Availability

The datasets used and/or analyzed during the present study are available from the corresponding upon request.

## References

[1] Son H., Son Y. J., Kim H., Lee Y. (2018). Effect of psychosocial interventions on the quality of life of patients with colorectal cancer: a systematic review and meta-analysis. *Health and Quality of Life Outcomes*.

[2] Cotrim H., Pereira G. (2008). Impact of colorectal cancer on patient and family: implications for care. *European Journal of Oncology Nursing*.

[3] Piwonka M. A., Merino J. (1999). A multidimensional modeling of predictors influencing the adjustment to a colostomy. *Journal of WOCN*.

[4] Costa A. L. S., Heitkemper M. M., Alencar G. P., Damiani L. P., Silva R. M. D., Jarrett M. E. (2017). Social support is a predictor of lower stress and higher quality of life and resilience in Brazilian patients with colorectal cancer. *Cancer Nursing*.

[5] Sharour L. A., Omari O. A., Salameh A. B., Yehia D. (2020). Health-related quality of life among patients with colorectal cancer. *Journal of Research in Nursing*.

[6] Hosseini Rafsanjani T., Arab M., Ravari A., Miri S., Safarpour H. (2017). A study on the effects of spiritual group therapy on hope and the mental and spiritual health of patients with colorectal cancer. *Progress in Palliative Care*.

[7] Trinquinato I., Marques R., Ticona S., Antonietti C., Costa A. (2017). Gender differences in the perception of quality of life of patients with colorectal cancer. *Investigación y Educación en Enfermería*.

[8] Itatani Y., Kawada K., Sakai Y. (2018). Treatment of elderly patients with colorectal cancer. *BioMed Research International*.

[9] Chen B. P., Awasthi R., Sweet S. N. (2017). Four-week prehabilitation program is sufficient to modify exercise behaviors and improve preoperative functional walking capacity in patients with colorectal cancer. *Supportive Care in Cancer*.

[10] Ommundsen N., Wyller T. B., Nesbakken A. (2018). Preoperative geriatric assessment and tailored interventions in frail older patients with colorectal cancer: a randomized controlled trial. *Colorectal Disease*.

[11] Beesley V. L., Vallance J. K., Mihala G., Lynch B. M., Gordon L. G. (2017). Association between change in employment participation and quality of life in middle‐aged colorectal cancer survivors compared with general population controls. *Psycho-Oncology*.

[12] Sineshaw H. M., Ng K., Flanders W. D., Brawley O. W., Jemal A. (2018). Factors that contribute to differences in survival of black vs white patients with colorectal cancer. *Gastroenterology*.

[13] Momen‐Heravi F., Babic A., Tworoger S. S. (2017). Periodontal disease, tooth loss and colorectal cancer risk: results from the nurses’ health study. *International Journal of Cancer*.

[14] Witte H., Handberg C. (2019). An assessment of survivorship care needs of patients with colorectal cancer: the experiences and perspectives of hospital nurses. *Journal of Clinical Nursing*.

[15] Walker J. G., Bickerstaffe A., Hewabandu N. (2017). The CRISP colorectal cancer risk prediction tool: an exploratory study using simulated consultations in Australian primary care. *BMC Medical Informatics and Decision Making*.

[16] Tuominen L., Ritmala-Castrén M., Nikander P., Mäkelä S., Vahlberg T., Leino-Kilpi H. (2021). Empowering patient education on self-care activity among patients with colorectal cancer—a research protocol for a randomised trial. *BMC Nursing*.

[17] Moug S. J., Bryce A., Mutrie N., Anderson A. S. (2017). Lifestyle interventions are feasible in patients with colorectal cancer with potential short-term health benefits: a systematic review. *International Journal of Colorectal Disease*.

[18] White P. M., Itzkowitz S. H. (2020). Barriers driving racial disparities in colorectal cancer screening in African Americans. *Current Gastroenterology Reports*.

[19] Guo R., Wang H. (2021). Analysis of lung imaging intelligent diagnosis system for nursing intervention of lung cancer patients’ quality of life. *Contrast Media and Molecular Imaging*.

[20] Liang H., Huang J., Tong J., Wang J. (2021). Application of rapid rehabilitation nursing in thoracic surgery nursing. *Journal of Healthcare Engineering*.

[21] Sussman J., Bainbridge D., Whelan T. J. (2018). Evaluation of a specialized oncology nursing supportive care intervention in newly diagnosed breast and colorectal cancer patients following surgery: a cluster randomized trial. *Supportive Care in Cancer: Official Journal of the Multinational Association of Supportive Care in Cancer*.

[22] Blanes-Vidal V., Baatrup G., Nadimi E. S. (2019). Addressing priority challenges in the detection and assessment of colorectal polyps from capsule endoscopy and colonoscopy in colorectal cancer screening using machine learning. *Acta Oncologica*.

[23] Yu S., Tang Y. (2021). Effects of comprehensive care on psychological emotions, postoperative rehabilitation and complications of colorectal cancer patients after colostomy. *American Journal of Tourism Research*.

[24] Odun-Ayo F., Reddy L. (2022). Gastrointestinal microbiota dysbiosis associated with SARS-CoV 2 infection in colorectal cancer: the implication of probiotics. *Gastroenterology Insights*.

[25] Ferini G., Tripoli A., Umina V. (2021). Radiation proctitis: the potential role of hyaluronic acid in the prevention and restoration of any damage to the rectal mucosa among prostate cancer patients submitted to curative external beam radiotherapy. *Gastroenterology Insights*.

[26] Forma A., Chilimoniuk Z., Januszewski J., Sitarz R. (2021). The potential application of allium extracts in the treatment of gastrointestinal cancers. *Gastroenterology Insights*.

